# Selling the product: Strategies to increase recruitment and retention of Spanish-speaking Latinos in biomedical research

**DOI:** 10.1017/cts.2018.314

**Published:** 2018-10-22

**Authors:** Scott D. Rhodes, Jorge Alonzo, Lilli Mann-Jackson, Amanda E. Tanner, Aaron T. Vissman, Omar Martinez, Rodrigo Rodriguez-Celedon, Jesus M. Garcia, Jorge E. Arellano Hall, Eunyoung Y. Song, Eugenia Eng, Beth A. Reboussin

**Affiliations:** 1 Department of Social Sciences and Health Policy, Wake Forest School of Medicine, Winston-Salem, NC, USA; 2 Wake Forest Clinical and Translational Science Institute, Program in Community Engagement, Winston-Salem, NC, USA; 3 Section on Infectious Diseases, Wake Forest School of Medicine, Winston-Salem, NC, USA; 4 Department of Public Health Education, University of North Carolina Greensboro, Greensboro, NC, USA; 5 Center for Health and Human Services Research, Talbert House, Inc, Cincinnati, OH, USA; 6 School of Social Work, Temple University, Philadelphia, PA, USA; 7 Department of Health Behavior, University of North Carolina Chapel Hill, Chapel Hill, NC, USA; 8 Department of Biostatistical Sciences, Wake Forest School of Medicine, Winston-Salem, NC, USA

**Keywords:** Hispanic/Latino, CBPR, recruitment, retention, health disparities

## Abstract

**Introduction:**

The Latino population in the United States is rapidly growing and faces profound health disparities; however, engagement of Latinos in biomedical research remains low. Our community-based participatory research partnership has recruited 2083 Spanish-speaking Latinos into 21 studies over 15 years. We sought to identify and describe the strategies we have used to successfully recruit and retain Spanish-speaking Latinos in research.

**Methods:**

We abstracted and analyzed data from archived study notes, progress reports, team meeting minutes, and in-depth interviews conducted annually from community-based participatory research partnership members. We used a nominal group process to refine and prioritize strategies.

**Results:**

Overall, 13 recruitment strategies and 12 retention strategies emerged. These strategies relied on the creativity and perseverance of the study team and partners.

**Conclusions:**

It is essential that we develop and disseminate effective recruitment and retention strategies that engage Latinos in biomedical research to reduce health disparities and promote health equity.

## Introduction

Hispanics/Latinos (referred to as Latino hereafter) constitute the largest ethnic minority in the United States, representing 18% of the nation’s total population or about 58 million people as of 2016 [1]. However, like some minority groups in the United States, including African Americans, Latinos experience numerous health disparities when compared with non-Latino whites. For example, compared with non-Latino whites, Latinos have higher incidence and prevalence of and/or higher mortality from obesity, cardiovascular disease, cancer, diabetes, human immunodeficiency virus (HIV) infection, sexually transmitted infections, chronic liver disease and cirrhosis, and hypertension and hypertensive renal disease. They also have decreased access to healthcare resources compared to non-Latino whites [2–5]. The National Institutes of Health requires the representation of minority racial/ethnic populations in sponsored human subject research to determine whether the intervention or therapy being studied affects members of minority populations and their subpopulations differently in order to reduce and not perpetuate health disparities [6]. Yet, to date, Latinos remain significantly underrepresented as participants in biomedical research [5].

Although adequate representation of Latinos has been expected within large clinical trials on, their numbers have been disappointingly small. For example, the small percentages of Latinos in the Antihypertensive and Lipid-Lowering Treatment to Prevent Heart Attack Trial (ALLHAT) [7], Systolic Pressure Intervention Trial (SPRINT) [8], and Action to Control Cardiovascular Risk in Diabetes (ACCORD) [9] have prevented a complete understanding of disease progression and treatment among Latinos and subgroups. Thus, there is a profound need to improve Latino engagement in research to ensure that research improves rather than perpetuates health disparities [10].

There are multiple factors that contribute to the underrepresentation of Latinos in biomedical research; among them are mistrust or concerns about being treated as “guinea pigs”; misconceptions about eligibility; fears of detention and deportation for those who lack proper immigration documentation; assumed connections between researchers and immigration officials among those who may be undocumented; stigma and discrimination related to being Latino, an immigrant, and/or a sexual and/or gender minority; concerns about confidentiality and privacy; and language barriers including limited bilingual and bicultural research staff and study materials [11–13].

Over the past 15 years, our community-based participatory (CBPR) research partnership in North Carolina has been developing and implementing strategies to overcome these barriers to engage Spanish-speaking Latinos in cross-sectional observational and longitudinal studies designed to explore and improve the health of this growing and vulnerable population. Given our successes, we sought to identify and describe the strategies we have used to recruit and retain Spanish-speaking Latinos, including subpopulations such as Latino men who have sex with men (MSM) and Latina transgender women who are often considered difficult for researchers to reach.

## Methods

Our partnership uses CBPR, which is based on the underlying principle that “outside” researchers, such as academic and government scientists, have limited understanding of the lived experiences of community members and cannot fully appreciate how social, cultural, political, and economic contexts affect members within a specific community. They may lack insights on what communities expect and how communities “get things done.” Thus, CBPR brings together diverse partners to engage in ongoing multidirectional dialog to improve each phase of the research process, including the identification of research needs and priorities; hypothesis generation; study design formulation; participant engagement; data collection, analysis, and interpretation; and dissemination of findings [14–16]. Long-term partnerships between Latino community members; representatives from community organizations, such as health departments, recreational Latino soccer leagues, and lesbian, gay, bisexual, and transgender (LGBT)-serving organizations; Latino and non-Latino business leaders and employees; and scientists from academic and government institutions have been critical to developing successful recruitment and retention strategies in our studies.

To date, our CBPR partnership has successfully recruited a total of 2083 Spanish-speaking Latino men and Latina women, including transgender women, to participate in 21 completed research studies. In each study, we met planned recruitment and retention benchmarks. Table 1 highlights each study, the number of participants enrolled, select demographics, the recruitment rate, and the follow-up period and retention rate, as applicable. Recruitment rates across observational studies ranged from 83% to 100%. Recruitment rates across longitudinal studies ranged from 90% to 100%.

**Table 1:** Details of Latino men and Latina women and transgender women involvement by project

Study title	Study number/funder	Study type	Sampling	Number of enrolled Latino/a participants	Select demographics	Recruitment rate*	Follow-up period	Retention rate
HIV prevention among Latino MSM: evaluation of a locally developed intervention; The HOLA en Grupos intervention [18]	U01PS001570/CDC	Group-level with intervention and comparison arms	Longitudinal	304 (100% of sample)	MSM (94%) and transgender women (6%); mean age=30; 62% from Mexico; 45% reported educational attainment <high school	93%	6 months	100%
Evaluating a social media intervention to increase HIV testing; the CyBER intervention [34]	R01MH092932	Social media intervention	Cross-sectional	69 (5% of sample)	MSM (100%); mean age=39, range: 18–60	UK	NA	NA
The impact of immigration enforcement by local officials on access to care among Latinos [35]	Robert Wood Johnson Foundation	Focus groups and in-depth interviews	Cross-sectional	83 (100% of sample)	Men (36%) and women (64%); mean age=37, range: 19–86; 60% from Mexico; 65% reported educational attainment of ≤ high school	98%	NA	NA
Partnership approach to reducing HIV disparities among Latino men; The HoMBReS Por un Cambio intervention [20]	R24MD002774	Lay health advisor intervention using intervention and delayed-intervention design	Longitudinal	258 (100% of sample)	Heterosexually active men (95%) and MSM (5%); recreational soccer-team members; mean age=27, range: 18–54; 76% from Mexico; 77% reported educational attainment of <high school	99%	12 months	82%
Partnership approach to reducing HIV disparities among Latino men; The HoMBReS Por un Cambio intervention [36]	R24MD002774	Individual in-depth interviews	Cross-sectional	5 (42% of sample)	Heterosexually active men who reported paying for sex from women or transgender women (n=4); Transgender woman who reported exchanging sex for money (n=1); men mean age=36; transgender woman mean age=28; all from Mexico	83%	NA	NA
Partnership approach to reducing HIV disparities among Latino men; The HoMBReS Por un Cambio intervention [37]	R24MD002774	Individual in-depth interviews	Cross-sectional	25 (100% of sample)	Women; mean age=33, range: 21–47; 84% reported educational attainment <high school; 84% unemployed	100%	NA	NA
Using CBPR to reduce HIV risk among immigrant Latino MSM: Photovoice with Latina transgender women [26]	R01MH087339	Photovoice	Longitudinal	9 (100% of sample)	Transgender women (100%); mean age=30, range: 22–45; 100% from Mexico	90%	6 months	100%
Using CBPR to reduce HIV risk among immigrant Latino MSM; the HOLA intervention [19, 38]	R01MH087339	Lay health advisor intervention with intervention and delayed-intervention arms	Longitudinal	189 (100% of sample)	MSM (82%) and transgender women (18%); mean age=30, range: 18–30; 75% from Mexico	94%	12 months	95% at 12 months
Exploring HIV prevention among African American, Latino, and White MSM [25]	NC Department of Health and Human Services	Focus groups	Cross-sectional	33 (38% of sample)	MSM and transgender women; mean age=27, range: 18–60; 61% from Mexico	97%	NA	NA
HIV among rural Latino MSM in the Southeast: Qualitative phase [39]	R21HD049282	Individual in-depth interviews	Longitudinal	21 (100% of sample)	MSM (90%) and transgender women (10%); mean age=31, range: 18–48; 86% from Mexico	100%	2 weeks	100%
HIV among rural Latino MSM in the Southeast: Respondent-driven sampling (RDS) phase [40]	R21HD049282	Respondent-driven sampling	Cross-sectional	190 (100% of sample)	MSM (84%) and transgender women (16%); mean age=25.5, range: 18–48; 79% from Mexico	100%	NA	NA
Trabajando Juntos: working for health disparity reduction among Latinos; the HoMBReS-2 intervention [17]	R21MH079827	Small-group with intervention and comparison arms	Longitudinal	142 (100% of sample)	Spanish-speaking, heterosexually active men	99%	3 months	98%
Trust and mistrust of evidence-based medicine among Latinos with HIV: elicitation interview phase [41]	107201-44-RGAT, amfAR: The Foundation for AIDS Research	Individual in-depth interviews	Cross-sectional	25 (100% of sample)	HIV-positive and negative men and women; mean age=38; and 80% from Mexico	93%	NA	NA
Trust and mistrust of evidence-based medicine among Latinos with HIV: quantitative phase [42]	107201-44-RGAT, amfAR: The Foundation for AIDS Research	Clinic-based cross-sectional sample	Cross-sectional	66 (100% of sample)	HIV-positive men and women; mean age=38; 71% heterosexual, and 86% from Mexico or Central America	80%	NA	NA
Use of prescription drugs obtained from nonmedical sources for STD treatment among Latinos: qualitative phase [43]	02885-08/CDC	Individual in-depth interviews	Cross-sectional	30 (100% of sample)	Men (56%), women (39%), and transgender woman (6%); mean age=39, range: 23–64; 69% from Mexico	93%	NA	NA
Use of prescription drugs obtained from nonmedical sources for STD treatment among Latinos: Respondent-driven sampling (RDS) phase [44]	02885-08/CDC	Respondent-driven sampling	Cross-sectional	164 (100% of sample)	Men (36%) and women (64%); mean age=34; 85% from Mexico	100%	NA	NA
CBPR on pesticide exposure and neurological outcomes for Latinos [45]	R01ES008739	Quantitative assessment and blood collection	Cross-sectional	100 (100% of sample)	Men (100%); farm workers; mean age=37, range: 19–68; 100% from Mexico; 45% reported educational attainment of ≤6 years	92%	NA	NA
HIV and community capacity among Latinos [46]	Wake Forest Venture Funds	Photovoice	Longitudinal	9 men (100% of sample)	Heterosexually active men (100%); mean age=24, range: 18–29; 100% reported educational attainment of ≤8 years	100%	3 months	100%
A lay health advisor approach to HIV and STD prevention; The HoMBReS intervention [47]	TS-1023/CDC	Lay health advisor intervention with intervention and delayed-intervention arms with urine collection	Longitudinal	222 (100% of sample)	Men (100%); recreational soccer-team members; mean age=30; 61% from Mexico; 53% reported educational attainment of ≤8 years; all self-identified as heterosexual; 6 reported MSM behavior in past 12 months	UK	18 months	80%
Sexual health among Latino men in NC [48]	Kellogg Foundation	Focus groups	Cross-sectional	70 (100% of sample)	Men (100%); mean age=29, range: 18–51	100%	NA	NA
HIV and community capacity among Latinos [49]	Wake Forest Venture Funds	Focus groups	Cross-sectional	74 (100% of sample)	Men (100%); mean age=22, range: 18–37; 75% from Mexico	100%	NA	NA

* Among those screened eligible.

AIDS, acquired immune deficiency syndrome; CBPR, community-based participatory research; CDC, Centers for Disease Control and Prevention; HIV, human immunodeficiency virus; MSM, men who have sex with men; NA, not applicable (e.g., as in not a longitudinal study); NC, North Carolina; STD, sexually transmitted disease; UK, unknown because denominator was unknown (e.g., as in a web-based study).

Retention rates varied by study and follow-up period. For example, the evaluation study of the HoMBReS-2 intervention retained 98% of Latino men (n=142) at 3-month follow-up [17]. The evaluation study of the HOLA en Grupos intervention retained 100% of Latino MSM and Latina transgender women participants (n=304) at 6-month follow-up [18]. The evaluation study of the HOLA intervention retained 95% of Latino MSM and Latina transgender women participants (n=189) at 12-month follow-up [19, 20].

To identify successful strategies for recruitment and retention, an ad hoc subcommittee of the CBPR partnership abstracted and analyzed data from archived study notes, progress reports, team meeting minutes, and in-depth interview data collected annually from CBPR partnership members [21]. The subcommittee then grouped identified strategies into recruitment or retention strategies and combined strategies as appropriate. They developed a list of preliminary strategies and conducted an adapted nominal group process, a structured variation of a small-group discussion to reach consensus [22], with the entire partnership to refine, prioritize, and interpret recruitment and retention strategies.

## Results

A total, 13 recruitment strategies and 12 retention strategies emerged (Tables 2 and 3).

**Table 2:** Strategies to increase recruitment of Latino men and Latina women and transgender women in research

1. Recruiters were members of the study community and highlighted their similarities to potential participants
2. Community connections were nurtured to build trust
3. Materials that demonstrated university and organization affiliations legitimized studies
4. Social networks and social media were harnessed
5. Study uniqueness was emphasized
6. Potential participants were not pressured to enroll
7. “Good will” and a sense of commitment to community were tapped into
8. Recruitment materials were concise, tailored, and engaging
9. Teasers and samples of incentives were provided
10. Adapting to potential participants’ changing schedules and offering assistance to overcome challenges were critical
11. Cash compensation was provided for participation
12. Participants were identified, enrolled, and consented in the field at times of day and in locations that were convenient for them
13. Flexible, persistent, and simultaneous use of multiple strategies was critical

**Table 3:** Strategies to increase retention of Latino men and Latina women and transgender women in research

1. The most convenient day, time, and location were used for study activities
2. Venues in which study activities occurred were familiar and comfortable
3. Multiple names/nicknames used by and extensive contact information of participants were collected
4. The study team played “host” at study activities to make each participant feel special
5. Cash compensation was provided for participation
6. Culturally congruent snacks, candy, and meals were provided during study activities, and holidays and birthdays were celebrated when they coincided with study activities
7. Teasers were used to entice participants to return for subsequent study activities
8. Participants were thanked for their participation and reminded of subsequent study activities via their preferred social media and telephone calls
9. Obstacles to complete study activities were identified and overcome
10. “Good will” a sense of community were tapped into
11. Framed graduation certificates were provided to participants at the end of their participation
12. Flexible, persistent, and simultaneous use of multiple strategies was critical

### Recruitment Strategies

1. *Recruiters were members of the study community and highlighted their similarities to potential participants*. Community members who were similar to the study community based on numerous characteristics and identities, including age, culture, language, gender identity, and sexual orientation, recruited participants. Similarities between recruiters and potential participants helped recruiters connect with potential participants; however, recruiters who reflected the population being recruited only in terms of race/ethnicity were not as successful as recruiters who also shared other characteristics and identities with the target community. For example, Latino gay men were more successful than well-intentioned and well-trained Latino heterosexual men in recruiting Latino MSM. Furthermore, having similar immigration experiences or being able to relate to immigration experiences also facilitated mutual understanding of concerns and priorities that could build connectedness between recruiters and participants and reduce recruitment barriers.

Identifying recruiters was not easy. They often were identified by word-of-mouth. In many cases, recruiters worked in factories or community organizations and did not have a biomedical or research background prior to becoming a recruiter. They did not have high educational attainment, but they cared about and were connected to the community, were passionate about the research topic, and were interested in learning about research.

2. *Community connections were nurtured to build trust*. Recruiters, other study team members, including the principal investigator (PI), and other members of the CBPR partnership established a presence in the community before attempting to recruit participants. They met with community organizations that offered services to Latinos to introduce themselves. They did not assume that representatives at community organizations were as interested in the research as they were; rather, they asked questions about the experiences of and challenges faced by organizations in serving Latino populations. They prioritized what the organization representatives prioritized, described the study in ways that would resonate with these representatives, discussed ways to work together, and brainstormed strategies to recruit participants.

Recruiters, other study team members, and other members of the CBPR partnership also supported the work of these organizations and agencies by facilitating trainings, such as conducting resume writing trainings for clients and volunteering in organization-sponsored events (health fairs and fundraisers, as examples). This service illustrated their commitment to the organizations and to the community. Furthermore, to nurture and maintain established trust, our partnership has shared outcomes of studies with communities by conducting empowerment theory-based community forums. These forums bring together community members, organization representatives, and other stakeholders to learn about the progress and outcomes of a study and develop next steps. After reviewing findings, the group responds to 4 empowerment-based trigger questions that move from concrete (“What do you see in these findings?” and “In what ways do these findings make/not make sense to you?”) to validate and/or refine results and interpretations to more action-oriented and next steps (“What can be done?” and “What can we do?”) [23]. The objectives of the forums are to share study findings with community members and partners and be inclusive of community perspectives and priorities.

3. *Materials that demonstrated university and organization affiliations legitimized studies*. Although it is well established that study logos are critical for branding [24], recruiters also carried university badges and business cards and wore shirts and jackets with university and organization logos when they were in the community. Recognizable logos of trusted partners, including university partners, built trust and overcame wariness of potential participants.

4. *Social networks and social media were harnessed*. Potential participants were more receptive to a study if participation was recommended by someone they knew who had participated in the study previously, and word-of-mouth was particularly successful if the person recommending the study was respected within the community. Also, potential participants were identified and recruited through advertising the study via social media including Facebook. For studies that recruited MSM and transgender persons, Global Positioning System (GPS)-based mobile applications (“apps”) that some use for sexual and social networking (e.g., A4A/Radar, badoo, Grindr, Growlr, Jack’d, and SCRUFF) were also particularly successful in reaching potential participants with information and answering initial queries about the study.

5. *Study uniqueness was emphasized*. Recruiters stressed that the study was specifically designed and developed in Spanish for the population for which they were recruiting by professionals who shared their characteristics (e.g., Latino, gay, and/or immigrant). For example, when recruiting for the HOLA en Grupos HIV prevention intervention for Latino MSM and Latina transgender women [18], recruiters mentioned to potential participants that no other HIV prevention intervention like it existed for these populations.

6. *Potential participants were not pressured to enroll*. Although recruiters wanted to engage potential participants in dialog to motivate their interest in a study, they did not focus exclusively on the study and their recruitment numbers. Instead, they focused on the potential participant and communicated in ways and at the pace that each potential participant seemed comfortable. Recruiters were also familiar with local resources and made referrals if they became aware of participants’ needs other than those addressed by the study (e.g., support related to immigration issues, mental health, or homelessness). This respectfulness and awareness of participants’ competing needs and priorities further helped to build trust and readied potential participants to hear about the study and how it might help them or their communities.

7. *“Good will” and a sense of commitment to community were tapped into*. Recruiters raised awareness of the health issue being studied and emphasized the importance of each study. For example, recruiters explained to potential participants that their participation would help their communities and they would be “game changers” and “pioneers.” Recruiters shared that findings from studies could lead to further research and future programming that might benefit their communities as well as potentially helping Latino communities in other parts of the United States and in Latin America.

8. *Recruitment materials were concise, tailored, and engaging*. Study-specific materials used were developed by members of the community for which they were intended for use. Materials included visually stimulating posters and brochures that very briefly described the study and featured pictures of the study team, previous participants (who provided media-use consent), and the incentives provided for participating in the study. These materials were displayed and distributed at recruitment locations, such as at soccer team meetings, soccer fields during practice and games, clubs and bars, house parties, community events (e.g., gay pride festivals and Latino cultural festivals), Latino community-oriented businesses [e.g., tiendas (grocers), hair salons, barbershops, radio stations, and restaurants], and in apartment complexes where large numbers of Latinos lived.

9. *Teasers and samples of incentives were provided*. Sample materials from studies were used as teasers to pique the interest of potential participants. Recruiters gave out rubber bracelets that included the study logo and telephone number and showed potential participants examples of incentives, including T-shirts and caps with the logo and a framed certificate of completion with staff and PI signatures. In addition, recruiters emphasized that participating in a study could provide the opportunity to make new friends or meet others with similar experiences to their own, if the study included a group activity (as in the case of data collection through focus groups [25] or photovoice [26] or participation in an intervention [18]).

10. *Adapting to potential participants’ changing schedules and offering assistance to overcome challenges were critical*. Recruiters accommodated potential participants’ changes of plans in terms of timing and location for in-person screening and/or enrollment. Recruiters were also trained to assist the potential participants overcome obstacles, such as a lack of transportation, that prevented them from screening and/or enrollment [27]. Transportation is a challenge in states, like NC, that do not provide driver’s licenses to those who are undocumented [28] and at the same time have less developed public transportation infrastructures. Furthermore, recruiters did not assume a 9 am to 5 pm work schedule. They met potential participants where and when potential participants were available, including on weekends and late night.

11. *Cash compensation was provided for participation*. After consenting to participate in a study, participants were reimbursed with cash for their effort, transportation costs, and time. We have learned from participant feedback that cash is a preferable because it is perceived as more immediate and does not present real or perceived challenges for use compared with other formats such as gift cards or credit cards.

12. *Participants were identified, enrolled, and consented in the field at times of day and in locations that were convenient for them*. Rather than asking potential participants to come to study project offices, either at community organization and university offices, to discuss participation and complete enrollment and data collection, we met with potential participants in the field and completed enrollment and data collection at the same time whenever possible. Potential participants chose a variety of places (e.g., parking lots, fast food restaurants, Latino-owned stores, soccer fields, outside of parties and clubs/bars, and in or outside their homes). Recruiters also used their own cars as quiet and convenient locations for enrollment and data collection.

13. *Flexible, persistent, and simultaneous use of multiple strategies was critical*. We did not assume that one strategy would work to recruit all potential participants, and our partnership met frequently throughout the entire recruitment phase of each study to brainstorm new recruitment strategies, analyze successes and challenges of strategies, and tailor strategies to potential participant priorities. Recruiters were perseverant and creative and implemented multiple different strategies simultaneously.

### Retention Strategies

1. *The most convenient day, time, and location were used for study activities*. Study activities, including data collection and intervention sessions, were scheduled on the day of the week and at the time of day that participants were available. The locations of study activities were also selected to ensure ease in accessing each location (e.g., proximity to major highways, easily recognizable landmarks, and availability and ease of parking).

The study team also collected follow-up data in a variety of settings, such as before participants met their friends at clubs, before or after soccer team practice or matches, at parties and social gatherings (including outside a baby shower that was being held for an MSM participant and his female partner), and at workplaces, as examples. It was not unusual for members of the study team to collect follow-up data from participants when participants got off shift work as late as 2:30 am.

2. *Venues in which study activities occurred were familiar and comfortable*. We did not ask participants to come to our universities or to the offices of community organization partners for study activities. Rather, we used spaces that were most familiar to or comfortable for participants. Group activities, for example, were held in hotel conference rooms or in private rooms in Latino-owned restaurants, which offered privacy, a sense of security, and a feeling of community, and where we knew the staff were friendly to sexual and gender identity minorities, when relevant.

3. *Multiple names/nicknames used by an extensive contact information of participants were collected*. Many participants in our studies use multiple names, nicknames, or aliases. To keep track of all participants, we created excel sheets or REDCap electronic data capture tools for each study with all names or aliases used by participants and various ways to contact them, including names and aliases used on social media, such as Facebook and GPS-based mobile apps, and the contact information of close friends and family members who could help locate the participants in the future. Social media, particularly Facebook, was effective for staying in touch with participants, especially gay, bisexual, and other MSM and transgender women participants [18].

4. *The study team played “host” at study activities to make each participant feel special*. Participants were made to feel comfortable from the moment they arrived to a study activity, whether data collection or intervention implementation. They were welcomed and thanked for taking time to participate. Often, some participants arrived earlier than others, and while waiting for a group activity to begin, the study team offered them snacks in addition to making “small talk.” The study team also played music or YouTube videos of songs by popular Latino singers and videos from popular Latino TV shows while waiting for others to arrive.

The study team also paid attention to each participant during group activities. They were careful to welcome and say goodbye to each participant, use their first names, and help engage those who were not engaged in conversations with other participants. Helping participants connect with other participants and build a sense of community were also critical.

5. *Cash compensation was provided for participation*. Cash compensation was provided to participants for their time. Again, our study team has learned that cash is a preferable form of compensation as described within recruitment strategies above.

6. *Culturally congruent snacks, candy, and meals were provided during study activities, and holidays and birthdays were celebrated when they coincided with study activities*. The study team created a friendly and celebratory atmosphere to make study activities enjoyable and participants feel special. Providing culturally congruent snacks, such as candy from Latino tiendas and restaurant-catered meals that many participants recognized from their countries of origin, helped to create a sense of connection between the study team and participants. Also, when holidays or participant or study team birthdays coincided with study activities, the study team brought decorations and food that corresponded to that particular celebration.

7. *Teasers were used to entice participants to return for subsequent study activities*. At the end of each study activity, and before participants departed, study staff gave a brief description of the next study activity. For example, to create interest and anticipation, intervention participants were provided brief previews of activities (e.g., DVD vignettes) that were part of the next session [17, 18, 20]. Participants were also reminded of the incentives they would be receiving and the process was designed to be interactive. For example, if the next session included the provision of t-shirts, participants were asked not only their preferred size but also color. If the next session included the distribution of graduation certificates, participants were asked what name they wanted printed on their certificate.

8. *Participants were thanked for their participation and reminded of subsequent study activities via their preferred social media and telephone calls*. Participants received personalized messages of appreciation the day after a study activity and friendly and encouraging reminders for subsequent activities, as relevant. Furthermore, if the subsequent study activity included incentives, the study team confirmed choices (e.g., preferred t-shirt size and color and name for graduation certificate) between study activities to ensure contact information remained current, remind participants of upcoming activities, and increase anticipation among participations.

9. *Obstacles to complete study activities were identified and overcome*. A number of challenges made it difficult for participants to complete study activities. For example, many participants had multiple jobs and some employers required participants to justify absences from work. Thus, for participants who wanted written documentation of their participation in a study, we wrote letters on university letterhead that the participants presented to their employer to request to be excused from work or to change their shift in order to attend and complete study activities. The letters stated that the participant was involved in a community project but did not specify the health outcome or population that the study focused on. Employers appreciated these letters and excused absences or altered the participant’s work schedule.

Some participants did not have driver’s licenses or access to a car, and all lived in areas where public transportation was limited. The study team also provided or coordinated transportation to study activities as needed. The time spent during car rides was an additional opportunity for participants to bond with each other and the study team member (if driving) and further build trust. These relationships promoted retention as well.

We also found that some participants moved out of the state or out of the country before they had completed study activities (e.g., follow-up data collection). In these cases, we completed data collection over the phone and sent compensation by money transfer services such as Western Union.

10. *“Good will” and a sense of commitment to community were tapped into*. The study team frequently reminded participants of the important role they were playing to potentially effect change not only in their local communities but also in other Latino communities, and stressed that retention was a contributing factor to study success.

11. *Framed graduation certificates were provided to participants at the end of their participation*. For most participants, receiving a graduation certificate was meaningful and served as an incentive to encourage retention. Participants were informed in advance and reminded frequently that they would receive a certificate at the end of their participation in a study. Many participants in our studies were recent immigrants and had not had the opportunity to complete school or receive other recognition; the certificates were highly valued. In group study activities, the distribution of these certificates became an event with some participants bringing and wearing a cap and gown to receive their certificate at “graduation” from the study.

12. *Flexible, persistent, and simultaneous use of multiple strategies was critical*. As we found with recruitment, we did not assume that any one strategy would work to retain all participants. Our partnership met frequently to brainstorm retention strategies, analyze retention successes and challenges, and tailor strategies to participant priorities. Study team members were perseverant and creative and implemented multiple different retention strategies simultaneously.

## Discussion

Although there is growing recognition of the need to engage Latinos, including those who are primarily Spanish-speaking, in biomedical research, the development of Spanish-language materials alone is insufficient to ensure the recruitment and retention of this growing population. The barriers to the participation of Latino participation in biomedical research are profound, and some barriers may be intensifying due to the current political context and ongoing anti-immigration sentiment at both state and federal levels [29, 30]. However, our CBPR partnership has found that barriers are not insurmountable. Based on our successes across 21 studies, we have identified 25 strategies that we have used to recruit and retain Latinos in research.

These strategies include building and nurturing trusting relationships with community organizations and potential (for recruitment) and current participants (for retention); focusing on each potential and current participant and respecting their priorities and needs; using culturally congruent materials and strategies that resonate with potential and current participants; and conducting screening, enrollment, and as much of the study activities as possible in the field, within the community, and in locations that are familiar, safe, and comfortable. Successful recruitment and retention also relied on the creativity of study team members and partners to determine what messages and strategies resonated with community members.

While we found that the use of university and organization affiliations and logos legitimized studies, that is not always the case within some communities [31, 32]. The affiliation of a trusted community-based, nonacademic partner organization may increase trust, at least initially, within communities where members have felt like they have been exploited by research. Study team members can also build trust by being present at community events. Cosponsoring community events such as street fairs, community festivals, church gatherings, and local forums; staffing a booth or table at events; serving on local boards; and conducting educational outreach aimed to reach community audiences are examples of strategies that can be used by study team members from universities to increase community trust. Participation in other nonresearch activities, such as volunteering with community organizations or serving on local health coalitions, by study team members further advances community trust.

Study team members also were flexible; they were ready to collect data at any time, including on weekends and late at night. They were committed to the research. All members of study teams, including recruiters, data collectors, and other study staff, understood the research questions that the study they were working on was designed to answer, and they understood that in order to answer these questions certain numbers of participants were required. In the case of longitudinal studies, they also understood that certain numbers of participants needed to be followed over time; they understood concepts of rigor, power, sampling, and bias, and how these related to the study they were working on.

Successful recruitment and retention also relied on infrastructures that supported novel strategies. For example, dialog with our study team and the Institutional Review Board of record was required at times when we wanted to use locally developed, innovative recruitment and retention strategies, including the use of social media, such as Facebook and GPS-based mobile apps, to communicate with participants; the provision of letters to employers regarding work absences of participants; and posters and brochures that featured pictures of the study team, previous participants, and the incentives provided in the study. Our university Controller’s Office also helped to establish accounting systems to track cash compensation in the field.

It is important to note that Latinos living in the United States represent a diversity of nationalities, cultures, and traditions. About three-quarters of Latinos are of Mexican, Puerto Rican, or Cuban origin. There are also sizable populations of Dominicans, Guatemalans, and Salvadorians. The health of these subpopulations differ depending on their country of origin. Increasing Latino engagement in biomedical research will generate better understandings of subpopulation differences. Moreover, there are Latinos who blend Spanish and English in their communications, and thus, recruitment and retention materials that include both Spanish and English may appeal. For example, a project in Philadelphia designed to test an HIV prevention intervention for Latino same-sex couples changed its name from “Conectando Latinos en Pareja” to “Connecting Latinos en Pareja” based on community member guidance [33]; the blending of Spanish and English held meaning for the community being recruited and retained.

It is essential that study teams continue to develop and disseminate effective strategies that can successfully engage Latinos in biomedical research to improve our ability to reduce health disparities and promote health equity. We outlined strategies found to be successful but fundamental to our success was ongoing participation of our CBPR partnership and iterative discussion about what worked, what did not work, and what should be tried to ensure high recruitment and retention rates.
